# Human Forebrain Organoid-Derived Extracellular Vesicle Labeling with Iron Oxides for In Vitro Magnetic Resonance Imaging

**DOI:** 10.3390/biomedicines10123060

**Published:** 2022-11-28

**Authors:** Chang Liu, Shannon Helsper, Mark Marzano, Xingchi Chen, Laureana Muok, Colin Esmonde, Changchun Zeng, Li Sun, Samuel C. Grant, Yan Li

**Affiliations:** 1 Department of Chemical and Biomedical Engineering, FAMU-FSU College of Engineering, Florida State University, Tallahassee, FL 32310, USA; 2 The National High Magnetic Field Laboratory, Florida State University, Tallahassee, FL 32310, USA; 3 High Performance Materials Institute, Florida State University, Tallahassee, FL 32310, USA; 4 Department of Industrial and Manufacturing Engineering, FAMU-FSU College of Engineering, Florida State University, Tallahassee, FL 32310, USA; 5 Department of Biomedical Sciences, College of Medicine, Florida State University, Tallahassee, FL 32310, USA

**Keywords:** human pluripotent stem cells, forebrain organoids, extracellular vesicles, nanoscale iron oxides, magnetic resonance imaging

## Abstract

The significant roles of extracellular vesicles (EVs) as intracellular mediators, disease biomarkers, and therapeutic agents, make them a scientific hotspot. In particular, EVs secreted by human stem cells show significance in treating neurological disorders, such as Alzheimer’s disease and ischemic stroke. However, the clinical applications of EVs are limited due to their poor targeting capabilities and low therapeutic efficacies after intravenous administration. Superparamagnetic iron oxide (SPIO) nanoparticles are biocompatible and have been shown to improve the targeting ability of EVs. In particular, ultrasmall SPIO (USPIO, <50 nm) are more suitable for labeling nanoscale EVs due to their small size. In this study, induced forebrain neural progenitor cortical organoids (iNPCo) were differentiated from human induced pluripotent stem cells (iPSCs), and the iNPCo expressed FOXG1, Nkx2.1, α-catenin, as well as β-tubulin III. EVs were isolated from iNPCo media, then loaded with USPIOs by sonication. Size and concentration of EV particles were measured by nanoparticle tracking analysis, and no significant changes were observed in size distribution before and after sonication, but the concentration decreased after labeling. miR-21 and miR-133b decreased after sonication. Magnetic resonance imaging (MRI) demonstrated contrast visualized for the USPIO labeled EVs embedded in agarose gel phantoms. Upon calculation, USPIO labeled EVs exhibited considerably shorter relaxation times, quantified as T_2_ and T_2_^*^ values, reducing the signal intensity and generating higher MRI contrast compared to unlabeled EVs and gel only. Our study demonstrated that USPIO labeling was a feasible approach for in vitro tracking of brain organoid-derived EVs, which paves the way for further in vivo examination.

## 1. Introduction

Human induced pluripotent stem cells (iPSCs) have been a desirable replacement for embryonic stem cells since Yamanaka’s breakthrough [1]. iPSCs have great potential in differentiating to cells and organoids such as cardiomyocytes [2,3], kidney organoids [4,5], brain organoids [6,7,8], neural progenitor/stem cells (NP/SCs) [9,10,11], etc. This differential potential is attractive in disease therapy; however, the tumorigenicity if iPSCs and their derivatives remains a serious concern for clinical applications [9,12,13,14]. Recently, researchers discovered that the therapeutic efficiency of conditioned medium from mesenchymal stem cells (MSCs) was comparable to that of MSC administration, and the primary reason for its therapeutic efficiency can be linked to the extracellular vesicles (EVs) [15,16,17], making EVs a promising substitute for cell therapy.

Upadhya et al. [10] derived NSCs from iPSCs, isolated and characterized NSC-EVs through small RNA sequencing, proteomics, in vitro macrophage assay, as well as in vivo mouse and rat models, proving the brain repair ability of those EVs. However, the NSCs were 2D cell cultures, and it is believed that 2D systems might be less effective than 3D systems, considering the 3D cultures have cellular organization and extracellular matrix that resemble physiological conditions [18,19]. For example, Yuan et al. [20] revealed that the conditioned medium from mesenchymal stem cells (MSCs) in a 3D dynamic culture outperformed the 2D monolayer culture in terms of reducing CD8+ cytotoxic T cell proliferation, rejuvenating senescent cells, and decreasing reactive oxygen species.

Due to their significant roles not only as therapeutic agents [21,22,23] but also as intracellular mediators [24], and disease biomarkers [25,26,27,28], EVs have become a scientific hotspot. However, the clinical applications of EVs are limited due to their poor targeting capabilities and low therapeutic efficacy after intravenous administration. Superparamagnetic iron oxide (SPIO) nanoparticles are biocompatible and have been shown to improve the targeting ability of EVs when an external magnet field is applied [29,30]. SPIOs transport EVs to specific body locations under magnetic fields, and they can be readily controlled by varying the intensity or magnetic field orientation [31]. Based on their diameters, SPIOs are classified as standard SPIO (>50 nm) or ultra-small SPIO (USPIO, <50 nm). In the two classifications, USPIO is more suitable for labeling nanoscale EVs due to their size. When tracking EVs in vivo, SPIOs combined with magnetic resonance imaging (MRI) is a promising method [32]. 

In the engineering of EV cargo, it is critical not to disrupt the integrity of the EV membranes during the labeling in order to preserve their physiological functions [33,34]. SPIOs have the ability to label cells, then the labeled EVs are produced and secreted by the cells [35]. However, cells without phagocytic ability have limited capacity in taking up SPIOs, especially at low concentration [32,35]. Alternatively, SPIOs could be loaded into EVs by incubation directly [36], which minimizes disturbance to the membranes of EVs. Vesicle labeling via electroporation is another approach for labeling EVs with SPIOs [37,38]. One more labeling method is sonication, which is popular in loading active pharmaceutical ingredients for drug delivery systems [39,40]. These latter two strategies are effective, but they raise concerns regarding the integrity of EVs due to the strong force introduced to create pores on the membranes [35]. 

In our previous work [35], NPC organoids were incubated with SPIOs, and EVs were isolated from the conditioned medium. Though SPIOs were detectable in the organoids, MRI images did not support the presence of SPIOs in the secreted EVs. Using 1–2 magnitude higher SPIO concentrations, Dabrowska et al. [41] found their existence in the EVs through TEM and MRI; however, the cytotoxicity of this concentration of SPIOs was not assessed. In a drug delivery study [42], researchers loaded doxorubicin into macrophage-EVs through cell incubation, EV incubation, or EV sonication, and they showed that at pH 8.0, both the EV incubation and sonication groups loaded 2-fold doxorubicin than the cell incubation group. Besides, the EV sonication method showed higher gemcitabine loading capacity than the EV incubation method [43]. Therefore, it is hypothesized that sonication of EVs with USPIOs could achieve a desired USPIO loading efficiency.

In this paper, iNPCo was derived from iPSCs, and characterized for several key markers of the organoids. Then, the isolated EVs from the iNPCo condition media were loaded with USPIOs in EVs by sonication for 30 s in total, followed by incubation at 37 °C for one hour. EVs with or without USPIOs were characterized in terms of size, concentration, morphology, and miRNA expression. Besides, in vitro MRI analysis was performed for the USPIO-labeled EVs verses unlabeled EVs, showing the T_2_ and T_2_^*^ contrast. This work could serve as a foundation for future injection of forebrain organoid-EVs in animal models such as ischemic stroke, which can be tracked by MRI.

## 2. Materials and Methods

### 2.1. Cortical Organoid Differentiation from hiPSCs

Undifferentiated human iPSK3 cells were seeded into Ultra-Low Attachment (ULA) 24-well plates (Corning Inc., Corning, NY, USA) at 3 × 10^5^ cells/well in differentiation medium composed of DMEM/F-12 plus 2% B27 serum-free supplement (Life Technologies, Carlsbad, CA, USA). iPSK3 cells were seeded in the presence of Y27632 (10 μM). After 24 h, Y27632 was removed and the formed embryoid bodies (EB) were treated with dual SMAD signaling inhibitors of 10 μM SB431542 (Sigma-Aldrich, St. Louis, MO, USA) and 100 nM LDN193189 (Sigma) over 7 days. Then, on day 8, the spheroids were treated with fibroblast growth factor (FGF)-2 (10 ng/mL, Life Technologies, Carlsbad, CA, USA) and cyclopamine (an Shh inhibitor, 1 μM, Sigma, St. Louis, MO, USA) for cortical differentiation for up to 21 days [44,45,46]. The cells were re-plated onto growth factor reduced Matrigel-coated surfaces for another 8 days.

### 2.2. Flow Cytometry

The re-plated spheroids were trypsinized. For marker detection, trypsinized cells were fixed with 4% paraformaldehyde (PFA) and permeabilized with 100% cold methanol, blocked with 5% FBS in PBS, then stained with the corresponding marker antibody (Supplementary Table S1) overnight. The secondary Alexa Fluor 488 or 586 antibody was later used, incubated for one hour, then removed and rinsed with PBS twice, and then taken for flow cytometry measurement. The cells were acquired with a BD FACSCanto II flow cytometer (Becton Dickinson, Franklin Lakes, NJ, USA) and analyzed against isotype control using FlowJo software.

### 2.3. EV Collection and Isolation

The conditioned media were collected from the cortical spheroid cultures. The culture media contained serum-free B27 supplement, which had minimal EV interference from the media. To isolate cortical spheroid-derived EVs, a differential ultracentrifugation method was performed. Briefly, the conditioned media were centrifuged at 500× *g* for 5 min at 4 °C. The supernatants were collected and centrifuged again at 2000 g for 10 min. The collected supernatants were then centrifuged at 10,000× *g* for 30 min. Next, EVs were isolated using an inexpensive polyethylene glycol (PEG)-based method as reported previously [47,48]. The collected supernatants were mixed with PEG solution (24% wt/vol in 1.5 M NaCl) at a 2:1 volume and incubated at 4 °C overnight. The mixtures were then centrifuged at a series of speeds (1000× *g* for 10 min, 2000× *g* for 10 min, and then 3000× *g* for 40 min). The purpose of these steps is to collect as much EV pellet as possible. The crude EV pellets were resuspended in PBS and then ultra-centrifuged at 100,000× *g* for 70 min. Purified EV pellets were resuspended in 100 µL PBS. 

### 2.4. Nanoparticle Tracking Analysis (NTA)

Nanoparticle tracking analysis (NTA) was performed on the isolated EV samples in triplicate to determine size distribution and particle concentration. NTA was performed on a Nanosight LM10-HS instrument (Malvern Instruments, Malvern, UK) configured with a blue (488 nm) laser and sCMOS camera [47]. The EV samples were diluted to 1000 fold in PBS. For each replicate, three videos of 60 s were acquired with the camera shutter speed fixed at 30.00 ms. To ensure accurate and consistent detection of small particles, the camera level was set to 13, and the detection threshold was maintained at three. The laser chamber was cleaned thoroughly with particle-free water between each sample reading. The collected videos were analyzed using NTA3.4 software to obtain the mode and mean size distribution, as well as the concentration of particles per mL of solution. Compared to the mean size, the mode size is usually a more accurate representation because the vesicle aggregates may affect the mean size.

### 2.5. Preparation of Ultra-Small SPIOs

Two types of SPIO were used in this study. 1. SPIO-1 (8–10 nm and 15–20 nm). The iron oxide nanopowder (Fe_3_O_4_, high purity, 99.5+%) with Stock #: US3230, CAS#:1317-61-9 was purchased from US Research Nanomaterials, Inc. (Houston, TX, USA) Iron oxide nanopowder was mixed with sterile water at a concentration varying from 1 µM to 10 µM. The solution was water bath sonicated to distribute nanopowder into the water. 2. SPIO-2 (5 nm) The SPIO was prepared in water by Sigma Aldrich (725331-5ML) (St. Louis, MO, USA) and diluted with EV solution to a final volume of 500 µL mixture with an end concentration of 0.1 to 0.5 mg/mL.

### 2.6. EV Labeling with Nanoscale Iron Oxides by Sonication

EVs were isolated from iNPCo media and analyzed by NTA for particle concentration. Roughly 1 × 10^10^ EVs were suspended in 100 µL of PBS for sonication. The SPIOs were sonicated with EVs at a final concentration of 0.5 mg/mL of SPIO in EV solution at a volume of 500 µL. Sonication was conducted for 5 cycles of 2 s on followed by 2 s off and completed 3 times for each cycle, with 2 min of rest in between each cycle (30 s total). The dial was 2 Watts. After sonication, EVs + SPIO were incubated for 1 h at 37 °C. The EV + SPIO solution was then further purified by PEG spinning to remove EVs with SPIO from free SPIO in the solution. The EVs were then collected with particle free PBS at 100,000× *g* for 30 min and resuspended in 100 µL of PBS for further analysis.

### 2.7. In Vitro MRI Sample Preparation

EVs labeled with SPIO or without any SPIO suspended in 100 µL of PBS were layered in 1% agarose gel, each with a particle concentration of 1 × 10^10^/mL. Initially, 2% agarose gel (VWR, Suwannee, GA, USA) was heated to 42 °C in a 50 mL centrifuge tube to liquefy the gel and subsequently mixed with PBS or EV + PBS to form a 1% gel. The agarose gel was layered in a 10 mm glass NMR tube in the following specific sequence: the 200 µL 1% blank gel layer on the bottom of the tube was followed by a 100 µL control layer of non-labeled EVs. A 150 µL 1% blank gel was then layered between the control and the 100 µL SPIO labeled EVs layer. Any extra layers of EVs + SPIO were separated by 1% blank gel layers and a 200 µL 2% gel cap was applied on top. The 10 mm tube was kept on ice during this process to allow each layer to solidify before the next layer was applied.

### 2.8. In Vitro MRI Analysis

All MRI experiments were performed at the 21.1-T, 900-MHz vertical MRI scanner at the National High Magnetic Field in Tallahassee, FL, USA [49]. The magnet was equipped with a Bruker Avance III console and scans were recorded using Paravision 5.1 (Bruker, Inc., Billerica, MA, USA). An NMR tube containing the agarose-EV samples was loaded into a 10 mm birdcage ^1^H coil tuned to 900 MHz. Following that, several scans were acquired to calculate T_2_, T_2_^*^, and T_1_ values, all of which were acquired at (50 µm)^2^ in-plane resolution and 0.5 mm slice thickness. In brief, T_2_ relaxation was assessed using a multi-slice, multi-echo (MSME) pulse sequence with an effective TR = 5 s and TE in 14 ms increments ranging from 14 to 112 ms. Four averages were acquired for a total scan time of 1.1 h. T_2_^*^ relaxation was measured using a multi-echo GRE sequence with TR = 5 s and TE = 3.5 to 62 ms in 6.5 ms increments; 4 averages were acquired in a 50 min total scan time. T_1_ relaxation was measured using an MSME with variable repetition time (TR = 450, 908, 1450, 2111, 2960, 4150, 6150 and 15,000 ms) and an effective TE = 14 ms with two averages for a total scan time of 3.7 h. The acquisition temperature was maintained at 28 °C.

Average signal intensities of a region of interest delineated for each sample layer were measured in ParaVision and the resulting intensity vs. time profiles were plotted in Prism GraphPad 9.2.0 (GraphPad Software, San Diego, CA, USA). For *T*_2_ and *T*_2_^*^, an exponential decay function was fitted to the data, and values were calculated from their respective *R*_2_ and *R*_2_^*^ according to:

$$T_{2}=\frac{1}{R_{2}}\;\;\;\;\;\;\;or\;\;\;\;\;\;\;T_{2}^{*}=\frac{1}{R_{2}^{*}}$$

*T*_1_ values were calculated in a similar manner from the *R*_1_ which was extracted using an exponential growth function fit to the saturation recovery data. 

### 2.9. microRNA RT-PCR Analysis

Total microRNA (miRNA) was isolated from different EV samples using the miRNeasy Micro Kit (Qiagen, Valencia, CA, USA) according to the manufacturer’s protocol. Reverse transcription was carried out using the commercial qScript miR cDNA synthesis kit (Quantabio, Beverly, MA, USA). The PerfeCTa^®^ Universal PCR Primer (QuantaBio, Beverly, MA, USA) has been designed and validated to work specifically with the PerfeCTa SYBR Green SuperMix using miRNA cDNA produced. The levels of miRs were determined with SNORD44 as a housekeeping gene for normalization of miR expression levels (Primer sequences are shown in Supplementary Table S2). Real-time RT-PCR reactions were performed on an Applied Biosystems Quantstudio 7 flex (Applied Biosystems, Foster City, CA, USA), using SYBR1 Green PCR Master Mix (Applied Biosystems, Foster City, CA, USA). The amplification reactions were performed as follows: 10 min at 95 °C, and 40 cycles of 95 °C for 15 s and 60 °C for 30 s, and 70 °C for 30 s. Fold variation in gene expression was quantified by means of the comparative Ct method: $2^{-(\Delta C_{t\;treatment}-\Delta C_{t\;control})}$, which is based on the comparison of expression of the target gene (normalized to the endogenous control) between the compared samples.

### 2.10. Transmission Electron Microscopy (TEM)

Electron microscopy imaging was used to confirm the morphology and size of EVs. Briefly, EV isolates were resuspended in 30 μL of filtered PBS. For each sample preparation, intact EVs (15 µL) were dropped onto Parafilm. A carbon coated 400 Hex Mesh Copper grid (Electron Microscopy Sciences, EMS) was positioned using forceps with the coating side down on top of each drop for 1 h. Grids were rinsed three times with 30 µL filtered PBS before being fixed in 2% PFA for 10 min (EMS, EM Grade). The grids were then transferred on top of a 20 µL drop of 2.5% glutaraldehyde (EMS, EM Grade) and incubated for 10 min. Samples were stained for 10 min with 2% uranyl acetate (EMS grade). Then, the samples were embedded for 10 min with a mixture of 0.13% methyl cellulose and 0.4% uranyl acetate. The coated side of the grids were left to dry before imaging on the Transmission Electron Microscope HT7800 (Hitachi, Janan). Image analysis was performed in ImageJ to determine the average sizes of EVs.

### 2.11. Statistical Analysis

The representative experiments were presented, and the results were expressed as [mean ± standard deviation]. To assess the statistical significance, one-way ANOVA or student’s *t*-test followed by Fisher’s LSD post hoc tests were performed. A *p*-value < 0.05 was considered statistically significant.

## 3. Results

### 3.1. In Vitro Characterizations of Forebrain Organoid−EVs

**Figure 1:**
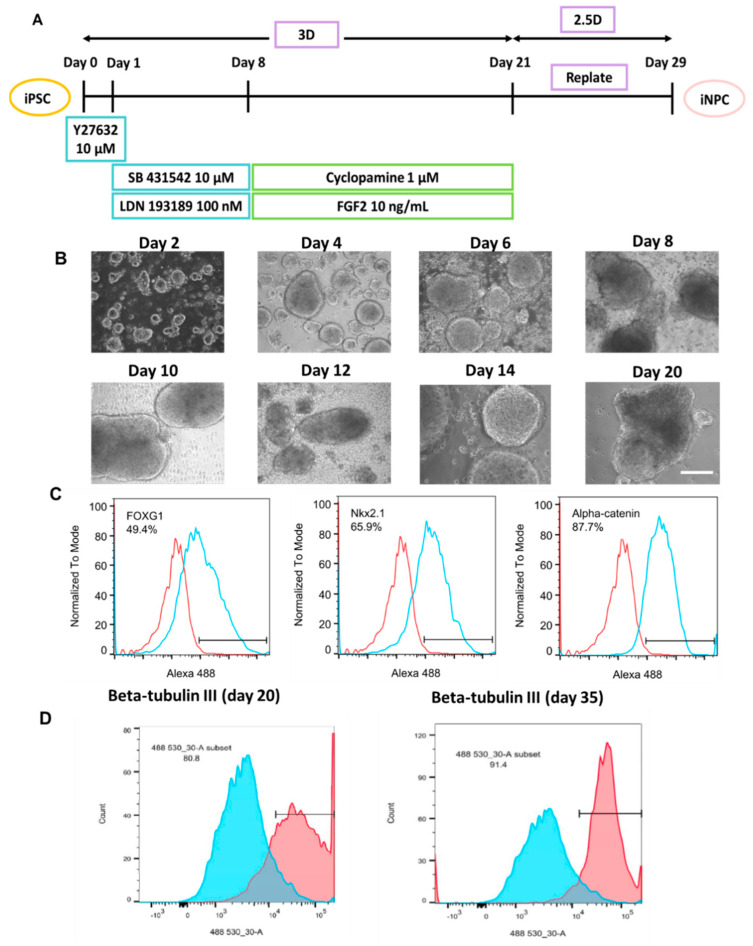
Characterization of cortical organoids derived from hiPSCs for EV collection. (**A**) Schematic illustration of cortical organoid differentiation from hiPSCs (**B**) Morphology of cortical organoids derived from hiPSCs; Scale bar: 200 µm. (**C**) Flow cytometry analysis of cells from cortical organoids for FOXG1, Nkx2.1, and α−catenin. Red line: negative control; Blue line: marker of interest. (**D**) Flow cytometry analysis of β-tubulin III. Blue: negative control.

The differentiation timeline of the forebrain organoids (iNPCo) was described in Figure 1A. The cells were cultured in low−attachment wells, facilitated with Y27632 in the first 24 h to promote aggregates formation. From day 0 to day 21, the cell aggregates grew into embryoid bodies and further developed into organoids (Figure 1B). Organoids harvested on day 21 were dissociated into single cells for markers staining and flow cytometry (Figure 1C). FOXG1 was moderately expressed (49.4%). Nkx2.1 expression level was high (65.9%). α−catenin had even higher expression (87.7%). β−tubulin III was highly expressed (80.8% and 91.4%) (Figure 1D).

**Figure 2:**
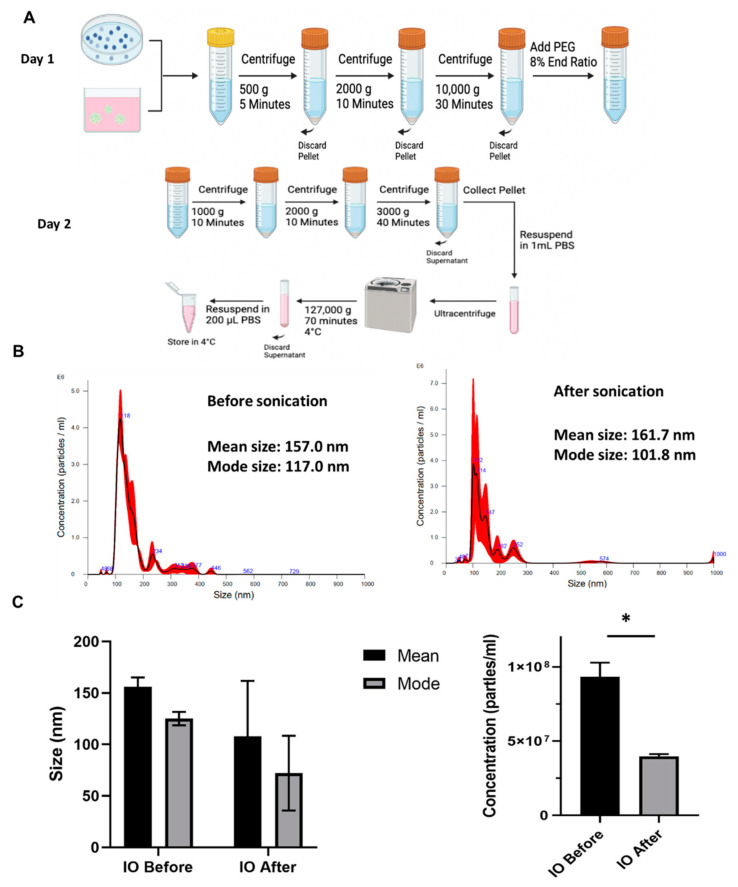
NTA for forebrain organoid-EV before and after sonication. (**A**) Schematic illustration of EV isolation by ExtraPEG method. The illustration was plot by Katelyn Sears. (**B**) Representative NTA EV size distribution, Before sonication; and after sonication. (**C**) EV size and concentration before and after sonication. * indicates *p* < 0.05.

The organoids were replated for 8 days, and the conditioned media were collected every two days. The process of EV isolation was shown in Figure 2A. The EV size distribution and particle counts were determined by NTA. Before the sonication, the mean size of EVs was 157.0 nm, and the mode size was 117.0 nm. After iron oxide labeling by sonication (the dial was 2 Watts), the EV mean size barely changed to 161.7 nm, while the mode size decreased slightly to 101.8 nm (Figure 2B,C, Supplementary Table S3). After sonication, the particle concentration per mL of conditioned media significantly decreased by about 60% (Figure 2C).

**Figure 3:**
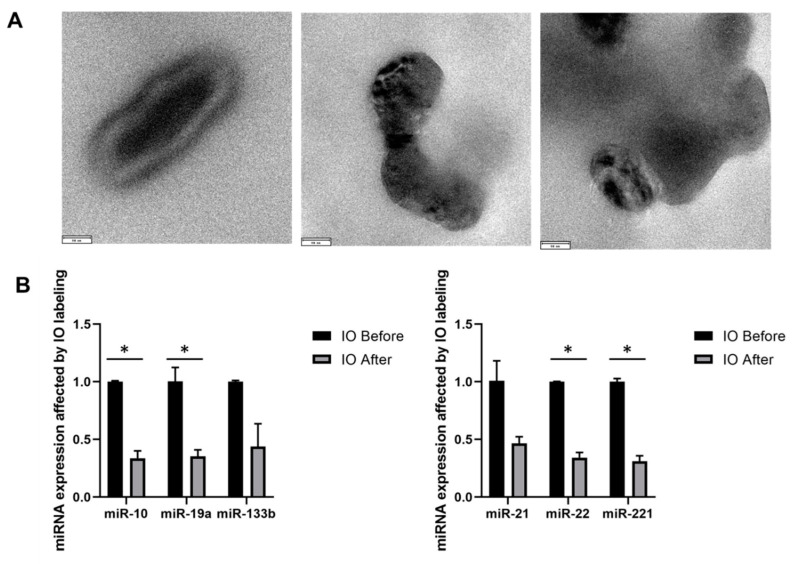
miRNA cargo for Organoid−EV before and after sonication and labeling. (**A**) Images of transmission electron microscopy (TEM) for EV morphology; Scale bar: 90 nm. (**B**) miRNA expression in the EVs before and after sonication determined by RT-PCR (*n* = 3), * indicates *p* < 0.05.

TEM imaging showed the double−layer cup−shape morphology of the EVs (Figure 3A). The darkness presented inside of the EVs indicated the successful involvement of USPIOs by the EVs. RNA was isolated from EVs before and after sonication. Then, the miRNA was reverse transcribed and amplified. The total RNA from unlabeled EVs was slightly lower than the labeled EVs (93.8 ng/μL vs. 126.8 ng/μL). The expression levels of 4 out of 6 miRNAs were significantly decreased in the after−sonication group, possibly due to the step loss of removing the extra USPIO through ultracentrifugation (Figure 3B).

### 3.2. In Vitro MRI of Labeled Forebrain Organoid−EVs

**Figure 4:**
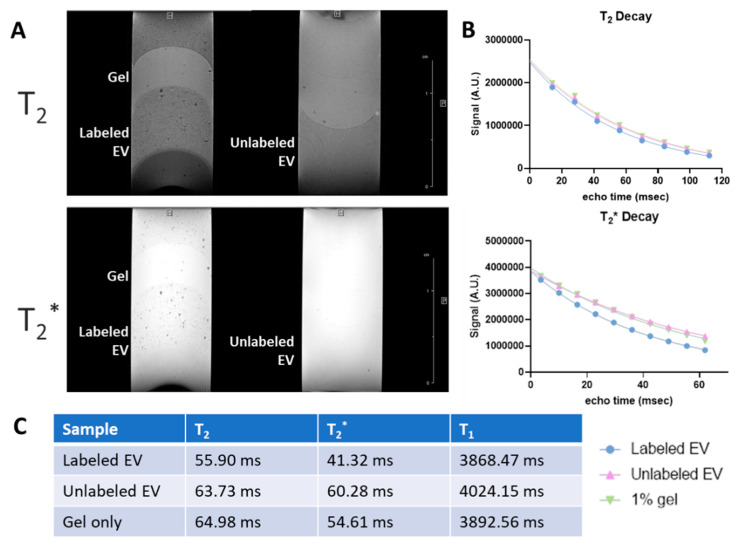
In vitro MRI for labeled brain organoid−EVs (15–20 nm). EVs sonicated with nanoscale iron oxides, 6 cycles: 30 s on/30 s off. Layered in agarose gel; (**A**) Preliminary results show the contrast of EVs containing iron oxides in MR images. (**B**) T_2_ and T_2_^*^ decay rates are demonstrated for labeled and unlabeled EV compared to gel only. (**C**) T_2_, T_2_^*^, and T_1_ values were extracted from their respective plots.

The feasibility of utilizing MRI to visualize and compare SPIO−labeled EVs was assessed. Agarose gel phantoms are commonly used to induce longitudinal and transversal relaxation rates comparable to that of in vivo tissues [50,51]. In vitro MRI analysis of labeled EVs embedded in 1% agarose gel was performed and compared between the different SPIO sizes and preparation methods. Figure 4 demonstrated minimal contrast within the layers of gel containing 15–20 nm SPIO−labeled EVs. The T_2_ and T_2_^*^ values were extracted from the average signal intensity and time profiles for each sample by fitting the respective data to a first exponential decay. As seen in Figure 4, only nominal changes in T_2_ and T_2_^*^ relaxation were established between sample layers, likely as a result of partial volume effects. This preparation method was repeated with similar outcomes using 8–10 nm of SPIO, as can be seen in Supplemental Figure S1. 

**Figure 5:**
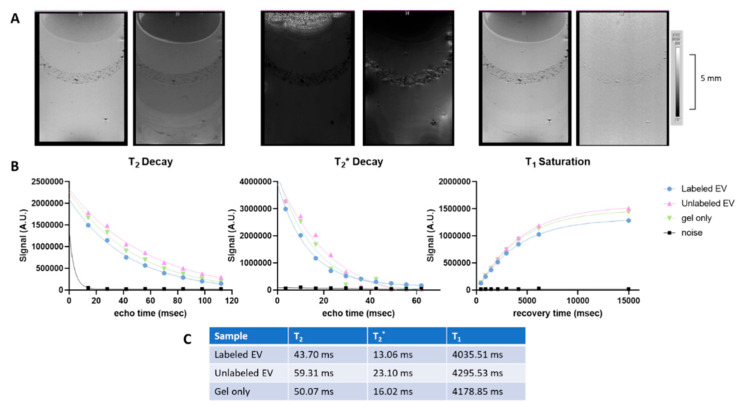
In vitro MRI for labeled brain organoid−EVs (15–20 nm) with a revised gel preparation method. (**A**) MR images demonstrated punctuated contrast in the labeled EVs layer compared to unlabeled EV. (**B**) T_2_ (left) and T_2_^*^ (middle) decay rates as well as T_1_ saturation (right) are demonstrated for labeled and unlabeled EV compared to gel only. (**C**) T_2_, T_2_^*^, and T_1_ values were extracted from their respective plots.

To minimize partial volume effects, the gel volume of EV layers was reduced, increasing the EV concentration within each layer. As demonstrated in Figure 5 (15–20 nm SPIO−labeled EVs), this resulted in slightly increased contrast, albeit still in a punctuated, heterogeneous manner, suggesting EV aggregation. When quantified, labeled EVs shortened T_2_ approximately 26.3% and T_2_^*^ 43.5% compared to unlabeled EV. Interestingly, unlabeled EV lengthened T_2_ and T_2_^*^ values by 15.5 and 30.6%, respectively, compared to gel only. Similarly, T_1_ saturation was also assessed, and values were extracted from the average signal vs. recovery time profile by fitting an exponential rise to maximum. As expected, T_1_ effects remained minimal with labeled EVs decreasing T_1_ only 6.4%. 

**Figure 6:**
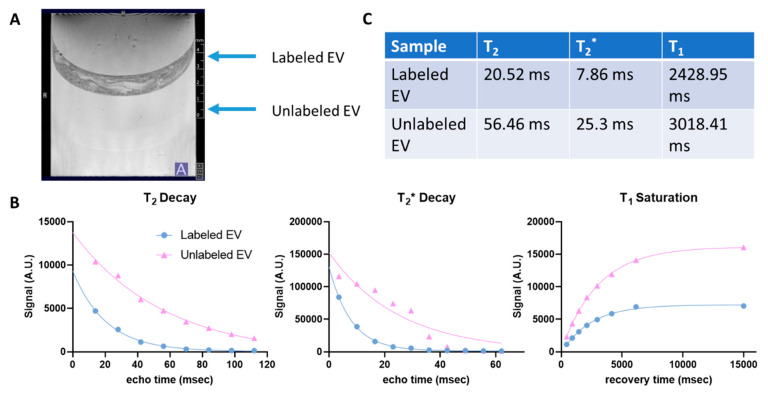
Enhanced in vitro MRI contrast for the labeled brain organoid−EVs (5 nm). (**A**) Labeled EVs were visualized as a mostly homogenous layer with significant contrast compared to unlabeled EVs. (**B**) Decay and saturation curves supported a strong MR contrast with (**C**) reduced T_2_, T_2_^*^ and T_1_ values for labeled EVs compared to their unlabeled counterparts.

A more homogenous layer of SPIO−generated contrast within the labeled EV layer was prepared by exposure to the SPIO of 5 nm at 0.5 mg/mL followed by ExtraPEG removal of free SPIO (Figure 6). Similar to previously, longitudinal and transverse relaxation were shortened within the SPIO-labeled EV layer. While the unlabeled EV layers exhibited comparable T_2_, T_2_^*^ and T_1_ values, as expected, this preparation resulted in 63.6, 68.1 and 19.5% reductions, respectively, for the labeled EVs. It is important to note that the region of interest used in the analysis avoided the central region of inhomogeneity caused by gel artifact. 

## 4. Discussion

This study derived iNPCo from iPSCs using a dual SMAD inhibition method [52]. The organoids showed forebrain neural identity due to the addition of FGF2 and sonic hedgehog (SHH) inhibitor cyclopamine [53]. β−tubulin III, a neuronal marker, was highly expressed in our iNPCo. α−catenin is a molecular link between β−catenin and the actin cytoskeleton, which is a crucial protein at cell junctions. It is also abundant in neural cells [54], and our iNPCo expressed α−catenin at a high level. During cerebral development, SHH promotes the hindbrain and posterior forebrain identity [53,55]. However, cyclopamine leads to a near complete inhibition of SHH signaling activity [53], and contributes to anterior characteristics, such as the expression of FOXG1 and Nkx2.1 [56,57], as examined in our study. However, applying 3D brain organoids in vitro culture systems to assess the therapeutic potential of the secreted EVs has not been well investigated [18,19]. 

Having derived iNPCo, the secreted EVs were isolated from the conditioned medium of the replated culture using the inexpensive ExtraPEG method [47]. Following that, the EVs were labeled with different sizes of iron oxides, including 15–20 nm, 8–10 nm, and 5 nm USPIO by sonication. Though the labeled EVs showed a smaller mode size, the size distribution did not change significantly, which demonstrates that the sonication method does not influence the particle size at a significant level. This result is similar to that of Nizamudeen et al.’s study [58], though they did not carry out EV labeling, but proved that low-power sonication resulted in an insignificant reduction in EV sizes when measured with NTA. However, it was significant when using other detection techniques such as dynamic light scattering and stochastic optical reconstruction microscopy. In our study, the EV concentration decreased by half, which may be due to the extra processing step following sonication. This step loss could be an issue that needs to be addressed in future studies.

The EV morphology and the internal USPIO were examined by TEM. The particles were found to be still double layered, with black dots inside, revealing the successful involvement of USPIO in EVs. The specific miRNA cargo after USPIO labeling was examined by RT-PCR. Four out of six tested miRNAs had decreased abundance after the labeling. Further optimizations of the labeling process to minimize the cargo loss during USPIO labeling are necessary. In many studies, USPIO were loaded into EVs through incubation with cells [35,59,60,61], which is less efficient due to the increasing number of cells thus would dilute the USPIO. Another method is electroporation [38,62], which causes a temporary breakage of the membrane, but is more effective than the incubation method. In our study, the sonication approach was employed, and was believed to have a high efficiency in incorporating USPIO due to the reorganization of the lipid membranes [63], but few studies reported USPIO labeling through sonication. The more popular applications for sonication include disease treatment and drug delivery, such as delivering RNAs [64], proteins [65], and chemicals [42]. However, the effects of labeling USPIO by sonication on EV cargos have not been extensively studied. Most USPIO studies focused on EV tracking through MRI without discussing the EV components; and most sonication studies (using EV as a carrier) focused on the molecules being delivered and the disease being treated while the EV native cargo was ignored. In our study, the EVs were labeled with USPIO by sonication, followed by one hour incubation at 37 °C for recovery [63]. The one−hour incubation restored the membrane microviscosity. According to the TEM images, labeled EVs showed elongated shape instead of the round shape which they usually be. This supported the idea of reorganization of the lipid membranes upon sonication. Besides, the TEM images demonstrated the integrity of the EVs, possibly due to flexibility of the lipid membranes. There are chances that the membrane integrity was damaged. The extent of the damage was unknown in this study, but the harm was minimized through incubation for recovery and stabilization. Though not quantitatively, it was reported that the exosome markers were maintained [63]. However, the loss of some miRNAs was observed in our study, leading to the application being selective. For example, miR−19a promotes cell proliferation [66] and angiogenesis [67], so when the intended application is to monitor USPIO loaded EVs for ischemic disease treatment, this labeling method might reduce the efficacy. When the aim is to recover neurite outgrowth via miR−133b [68], this sonication labeling approach could potentially fulfill the requirement. 

Visualization and tracking of EVs would be beneficial to understand their bio distribution potential and MRI is particularly suitable to this goal as it has already been employed successfully to track stem cells labeled with larger iron oxide particles in various disease models including ischemic stroke [69,70]. USPIOs have a predominant T_2_ effect, ultimately reducing signal and giving rise to the dark regions seen in the MR images provided in Figure 4, Figure 5 and Figure 6 and Supplementary Figure S1 [71]. Most commonly, T_2_^*^−weighted MRI is used to optimally visualize this contrast. Here, the effects of iron oxide size and EV preparation were investigated using MRI of the USPIO−labeled organoid−EVs embedded in tissue−mimicking gels. In the first preparation method, which consisted of 15–20 nm USPIO labeled EVs, partial volume effects contributed to low contrast within the labeled EV gel layers. This was corrected for in subsequent samples by decreasing the volume of gel within the EV layers. As a result, a more uniform layer of labeled EVs with higher MR contrast was generated, albeit in a punctate distribution suggesting potential aggregation of the EVs. In order to quantify the contrast generated by the USPIO, T_2_ and T_2_^*^ values were extracted from their respective exponential signal decays. Although more easily visualized compared to the previous preparation method, T_2_ and T_2_^*^ effects remained limited. Further optimization was pursued by reducing the USPIO size to 5 nm and incorporating the previously defined ExtraPEG method to remove free USPIO prior to embedding in the gel. This shortened the T_2_ and T_2_^*^ values resulting in significantly increased MRI contrast. Although the varying concentrations of labeled EVs require discretion in comparing between the first and second agarose gel preparations, the enhanced MRI contrast observed with the smaller USPIO size demonstrates the feasibility of labeling forebrain organoid−EVs using USPIO for non−invasive MRI toward potential animal study, such as ischemic stroke rat models. 

## 5. Conclusions

In this study, we generated forebrain organoid iNPCo from iPSC, and isolated EVs from the conditioned medium of the culture. The EVs were labeled with USPIO by the sonication method. Their size distribution did not change significantly, but their recovery and some of the miRNA cargo decreased after sonication. Though feasible, this labeling approach should be modified to reduce the loss of EV particles and EV cargo. MRI confirmed contrast visualization only for the USPIO labeled EVs embedded in agarose gel phantoms. USPIO labeled EVs exhibited shorter T_2_ and T_2_^*^ values compared to unlabeled EVs and gel only. Our study demonstrated that USPIO labeling was a practical strategy for in vitro tracking of EVs, which paves the way for further in vivo examination.

## Data Availability

Available upon request.

## Supplementary Materials

The following supporting information can be downloaded at: https://www.mdpi.com/article/10.3390/biomedicines10123060/s1, Figure S1: In vitro MRI for labeled brain organoid-EVs (8-10 nm); Table S1: A list of antibodies; Table S2: Primer sequences for microRNAs by RT-PCR analysis; Table S3: Nanoparticle tracking analysis (NTA) for the EVs used for in vitro MRI.
