# Supplementary material for: Human Forebrain Organoid-Derived Extracellular Vesicle Labeling with Iron Oxides for In Vitro Magnetic Resonance Imaging
Source: Biomedicines. 2022 Nov 28;10(12):3060. doi: 10.3390/biomedicines10123060 (PMC9775717; doi:10.3390/biomedicines10123060)
Supplement: Supplementary file 1 [file biomedicines-10-03060-s001.zip › biomedicines-2035930-supplementary.pdf]

Supplementary Materials

Human Forebrain Organoid-derived Extracellular Vesicle Labelling with Iron Oxides for In Vitro Magnetic Resonance Imaging

Chang Liu[1, a], Shannon Helsper[1,2, a], Mark Marzano[1], Xingchi Chen[1,3], Laureana Muok[1], Colin Esmonde[1], Changchun Zeng[3,4], Li Sun[1,5], Samuel C. Grant[1,2,*], Yan Li[1, *]

[1]Department of Chemical and Biomedical Engineering, FAMU-FSU College of Engineering, Florida State University, Tallahassee, Florida, USA
[2]The National High Magnetic Field Laboratory, Florida State University, Tallahassee, Florida.
[3]High Performance Materials Institute, Florida State University, Tallahassee, Florida.
[4]Department of Industrial and Manufacturing Engineering, FAMU-FSU College of Engineering, Florida State University, Tallahassee, Florida, USA
[5]Department of Biomedical Sciences, College of Medicine, Florida State University, Tallahassee, Florida, USA

[a]These two authors contribute equally to this work

Supplementary Figure S1. *In vitro* MRI for labeled brain organoid-EVs (8-10 nm). EVs labeled with 8-10 nm USPIO were embedded in 1% agarose gel. (A) Representative T_2^*-weighted MRI depicting minimal contrast generated from the labeled EVs. The large blooming artifact likely resulting from an aggregation of USPIO was avoided in subsequent image analysis. (B) T_2, T_2^* and T_1 signal intensity profiles were used to extract. (C) T_2, T_2^* and T_1 values.

A

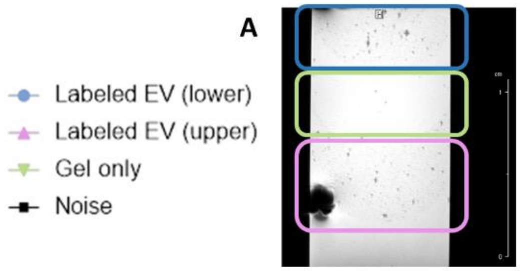

C

Sample	T_2	T_2^*	T_1
Labeled EV (lower)	42.74 ms	36.26 ms	4578.76 ms
Labeled EV (upper)	45.64 ms	26.77 ms	4589.26 ms
Gel only	47.64 ms	38.51 ms	4430.66 ms

B

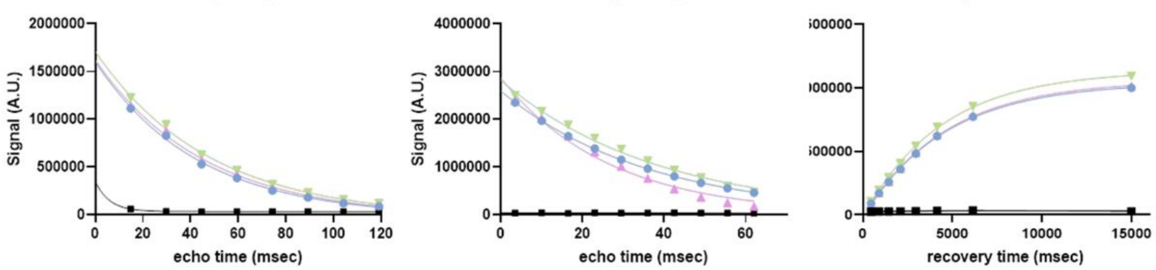

Supplementary Table S1. A list of antibodies.

Cells	Primary Antibody	Origin/ Isotype	Supplier/ Cat#	Dilution
Neural cells	FOXG1	Rabbit IgG	ThermoFisher, PA5-26794	1:100
	β-tubulin III	Mouse IgG_1	Millipore, MAB1637	1:200
	Nkx2.1	Rabbit IgG	Thermal Fisher, PA5-25940	1:200
Cell-cell adhesion	a-catenin	Mouse-IgG_1	Sigma, A7811	1:100
Secondary	Alexa 488, goat anti-mouse IgG_1	-	Life Technologies, A-21121	1:200
	Alexa 488, goat anti-rabbit IgG	-	Life Technologies, A-11034	1:200

Supplementary Table S2. Primer sequences for microRNAs by RT-PCR analysis.

ID	Human mirna sequence	Primer Sequence (5'-3')
hsa-miR-21-5p	UAGCUUAUCAGACUGAUGUUGA	GCTAGCTTATCAGACTGATGTTGAAA
hsa-miR-22-3p	AAGCUGCCAGUUGAAGAACUGU	CGAAGCTGCCAGTTGAAGAAC
hsa-miR-19a-3p	UGUGCAAAUCUAUGCAAAACUGA	TGTGCAAATCTATGCAAAACTGA
hsa-miR-221-3p	AGCUACAUUGUCUGCUGGGUUUC	GCGAGCTACATTGTCTGCTG
hsa-miR-221-5p	ACCUGGCAUACAAUGUAGAUUU	GCACCTGGCATACAATGTAGA
hsa-miR-133b	UUUGGUCCCCUUCAACCAGCUA	GTTTGGTCCCCTTCAACCA
hsa-miR-10a-5p	UACCCUGUAGAUCCGAAUUUGUG	ACCCTGTAGATCCGAATTTGTG
SNORD44	CCUGGAUGAUGAUAAGCAAAUGCUGACUGAACAUGAAGGUCUUAAUUAGCUCUAACUGACU	GCAAATGCTGACTGAACATGAA

Supplementary Table S3. Nanoparticle tracking analysis (NTA) for the EVs used for *in vitro* MRI. EVs are suspended in 100 µL PBS, NTA was conducted by 1:1000 dilution. So the total number of EVs is around 1.25×10^{11} particles in 100 µL

Concentration/mL	Mode (nm)	Mean (nm)	SD (nm)
1.34e9	125	187	72
1.25e9	124	183	63
1.15e9	144	198	107
